# DDA1, a novel oncogene, promotes lung cancer progression through regulation of cell cycle

**DOI:** 10.1111/jcmm.13084

**Published:** 2017-02-17

**Authors:** Lin Cheng, Qianmei Yang, Can Li, Lei Dai, Yang Yang, Qingnan Wang, Yu Ding, Junfeng Zhang, Lei Liu, Shuang Zhang, Ping Fan, Xun Hu, Rong Xiang, Dechao Yu, Yuquan Wei, Hongxin Deng

**Affiliations:** ^1^ State Key Laboratory of Biotherapy/Collaborative Innovation Center for Biotherapy West China Hospital Sichuan University Chengdu Sichuan China; ^2^ Core Facility of West China Hospital Sichuan University Chengdu Sichuan China; ^3^ Department of Medical Oncology Cancer Center West China Hospital Sichuan University Chengdu Sichuan China; ^4^ Huaxi Biobank West China Hospital Sichuan University Chengdu Sichuan China; ^5^ Department of Immunology Nankai University School of Medicine Tianjin China

**Keywords:** DDA1, cell proliferation, cell cycle, cyclins

## Abstract

Lung cancer is globally widespread and associated with high morbidity and mortality. DDA1 (DET1 and DDB1 associated 1) was first discovered and registered in the GenBank database by our colleagues. DDA1, an evolutionarily conserved gene, might have significant functions. Recent reports have demonstrated that DDA1 is linked to the ubiquitin–proteasome pathway and facilitates the degradation of target proteins. However, the function of DDA1 in lung cancer was previously unknown. This study aimed to investigate whether DDA1 contributes to tumorigenesis and progression of lung cancer. We found that the expression of DDA1 in normal lung cells and tissue was significantly lower than that in lung cancer and was associated with poor prognosis. DDA1 overexpression promoted proliferation of lung tumour cells and facilitated cell cycle progression *in vitro* and subcutaneous xenograft tumour progression *in vivo*. Mechanistically, this was associated with the regulation of S phase and cyclins including cyclin D1/D3/E1. These results indicate that DDA1 promotes lung cancer progression, potentially through promoting cyclins and cell cycle progression. Therefore, DDA1 may be a potential novel target for lung cancer treatment, and a biomarker for tumour prognosis.

## Introduction

DDA1 was first discovered by our colleagues through the serological analysis of recombinant cDNA expression libraries (SEREX) technique as a gene encoding a 1086‐bp cDNA and a 309‐bp open reading frame 1. DDA1 encodes an 11‐kD protein with 102 amino acid residues. DDA1 orthologs share 82–92% identity and include those of Arabidopsis, invertebrates and vertebrates 2, 3. We thus suggested that DDA1 could have a very important biological function across these species. DDA1, together with DET1 and DDB1, forms the DDD complex, which is recruited to specific UBE2E (E2 ubiquitin ligase) enzymes such as UBE2E1, UBE2E2 and UBE2E3 to form DDD‐E2 complexes 3. Components of the DDD‐E2 complexes provide a platform for interaction with Cullin4A (Cul4A) and beta‐transducing (also called WD40) repeat proteins, which indicated that this complex might be involved in ubiquitination and subsequent proteasomal degradation of target proteins 2, 4. Moreover, DDA1 was shown to be a core subunit of multiple Cul4‐based E3 ligases (CRLs) and could regulate CRL4s 5. In addition, DDA1 was also shown to interact with oncoproteins such as EIF3S10, PSAP and ACTN4 6. These results indicate that DDA1 might be involved in tumour formation and progression.

Lung cancer is the leading cause of cancer‐related mortality in the United States, accounting for more deaths than breast, prostate, colon and pancreatic cancers combined 7. This disease is often diagnosed at a more advanced stage and has poor prognosis. The combination of platinum‐based drugs and third‐generation anticancer drugs or molecular targeted therapies has been used for its clinical treatment 8. However, drug resistance leads to cancer recurrence. Therefore, the development of more effective molecular targets is important to improve current therapies for lung cancer treatment. Whether DDA1 has prognostic or therapeutic value in patients with lung cancer has not been previously assessed.

Loss of growth control is a hallmark of all cancers 9, including lung cancer. Major regulatory events leading to cell proliferation occur in the G1 phase, including the uncontrolled expression of cyclins. Cyclin E1, one of the most important cyclins, specifically drives the G1/S‐phase transition during this process. Cyclin E1 binds to and activates cyclin‐dependent kinase (CDK) 2, leading to phosphorylation of downstream substrates that control the initiation of DNA replication and other S‐phase events 10. Up‐regulation of cyclin E1 has been found in a variety of human cancers including breast, ovarian, colorectal and lung 10, 11, 12, 13, 14. Furthermore, D‐type cyclins are also important in cancer, as they represent the ultimate downstream targets of many oncogenic pathways 15, 16. D‐cyclins, binding and activating CDK4 and CDK6, phosphorylate the retinoblastoma tumour suppressor protein (pRB), and pRB‐like p107 and p130 proteins, leading to activation of E2F transcription factors 17. E2Fs then activate several downstream target genes that are required for the entry of cells into the DNA synthesis (S) phase 17. Amplification of individual cyclin D genes and overexpression of their encoded proteins have been documented in a large proportion of human cancers 18.

These results motivated us to investigate whether DDA1 participates in lung cancer tumorigenesis and tumour progression through the regulation of cyclins and cell cycle progression and thus could serve as a prognostic biomarker for lung cancer. Here, we demonstrated that DDA1 levels were increased in both lung adenocarcinoma and lung squamous cell carcinoma and that high expression of DDA1 was associated with poor prognosis. DDA1 promoted lung cancer cell proliferation, increased cell cycle progression *in vitro* through G1/S transition and S‐phase acceleration and regulation of cyclins. In addition, inhibition of DDA1 was shown to suppress tumorigenesis *in vivo* in a subcutaneous xenograft mouse model. Taken together, these results indicate that DDA1 promotes the progression of lung cancer by regulating the cell cycle, especially S phase, and cyclins such as cyclin D1/D3. DDA1 could be a powerful indicator of tumour prognosis in patients with lung cancer.

## Materials and methods

### Cell culture, transfection and plasmids

MRC‐5, NCI‐H292, NCI‐H526, 95‐D, NCI‐H441, NCI‐H358, A549, NCI‐H1299, Calu‐1, NCI‐H460, SPC‐A1, NCI‐H1975, NCI‐H69, NCI‐H446, NCI‐H1993 and NCI‐H2228 cell lines were obtained from the American Type Culture Collection (ATCC, Manassas, VA, USA). Cells were cultured in RPMI‐1640 (Gibco, Long Sheng Industry Park, Beijing, China) with 10% FBS (Gibco, Auckland, NZ, USA) and 1% penicillin and streptomycin with humidity at 37°C and 5% CO_2_. Cells were transfected by X‐tremeGENE HP DNA Transfection Reagent (Roche, Indianapolis, IN, USA). Plasmids pcDNA3.1(+) (Mock), pcDNA3.1(+)‐DDA1 (DDA1), pRNA‐U6.1‐CTL (shMock) and pRNA‐U6.1‐shDDA1 (shDDA1) were purchased from GenScript (Nanjing, China). All shRNA sequences are shown in Table S1.

### Tissue microarrays and immunohistochemistry (IHC)

Tissue microarrays containing FFPE (formalin‐fixed paraffin‐embedded) samples of lung cancer, adjacent tissue and normal lung tissues were purchased from US Biomax, Inc. (Rockville, MD, USA, LC10012, *n* = 100; T047, *n* = 18). Tissue microarrays with survival data were purchased from Shanghai Outdo Biotech CO. LTD. (Shanghai, China, HLug‐Ade150Sur‐02, *n* = 150; HLug‐Squ150Sur‐02, *n* = 150). The institutional review board approved the use of de‐identified samples; informed consent was obtained from all patients. A total of 418 tissues were analysed for DDA1 expression by IHC according to the manufacturer's recommendations (Vector Lab Inc., Burlingame, CA, USA). IHC scores were calculated as previously described 19.

### Quantitative PCR (qPCR), western blotting and immunofluorescence

qPCR, Western blotting and immunofluorescence were performed as described previously 20. For 5‐bromo‐2′‐deoxyuridine (BrdU) staining, Cells were probed by BrdU incorporation for 30 min., and then, cells were fixed and treated with 1.5 M HCl for 30 min. at room temperature and washed before blocking. Primers used for qPCR are summarized in Table S2. Antibodies are provided in supplemental materials.

### *In vitro* cell growth and colony formation assay

For cell growth assays, transfected cells were seeded at 2 × 10^3^ cells per well and six wells for each group in 96‐well plates. A Cell Counting Kit‐8 (Dojindo, Shanghai, China) was used, and absorbance was measures at 450 nm for each well at different time‐points using a microplate reader (Thermo fisher scientific, Waltham, MA, USA). For colony formation assays, transfected cells were plated at 500–1000 cells per well and three wells for each group into six‐well plates and cultured for approximately 14 days, followed by crystal violet staining.

### Flow cytometry assay for cell cycle

Cells were collected and fixed with 70% ethanol. The cell pellet was then treated with 50 μg/ml propidium iodide (PI, Sigma‐Aldrich, St. Louis, MO, USA) containing 0.1 mg/ml RNase A (Sigma‐Aldrich) and 0.1% Triton X (Sigma‐Aldrich), which was followed by flow cytometric analysis (BD Biosciences, San Jose, CA, USA). Data were analysed using FlowJo 9.1 software (Tree Star Inc., Ashland, OR, USA).

### Cell synchronization and cell cycle analyses

For thymidine and nocodazole blocking and release experiments, cells were treated with 2 mM thymidine (Sigma‐Aldrich) for 24 hrs and released for 9 hrs, followed by 200 ng/ml nocodazole (Sigma‐Aldrich) for 12–18 hrs. For cell cycle analyses, processing was performed as described previously herein.

### Xenograft tumours in nude mice

Lung cancer cells A549/LV‐mock, A549/LV‐DDA, H1299/LV‐shMock and H1299/LV‐shDDA1 (1 × 10^7^ cells in 100 μl of RPMI‐1640) were injected subcutaneously into the flanks of female BLAB/c nude mice (5 week old, Vital River Laboratories (VRL), China). Tumour size was determined by collecting length and width with a sliding caliper every 3 days, and calculating the tumour volume (mm^3^) as length × (width)^2^ × 0.52. When mice were killed, tumours from each animal were collected, weighed and used for histopathological studies and Western blotting. Procedures involving animals conformed to the guidelines of the Institutional Animal Care and Use Committee of West China Hospital, Sichuan University.

### Statistical analysis

A chi‐square test or Fisher's exact test was used to compare the differences in categorical variables. A Student's *t*‐test or an one‐way analysis of variance was used to analyse differences in continuous variables. A two‐way anova analysis, Huynh–Feldt correction and Tukey's range test were used to analyse tumour volumes. Kaplan–Meier analysis with log‐rank tests was used to evaluate overall survival. SPSS 22.0 statistical software (SPSS Inc., Chicago, IL, USA) was used to analyse all data. *P* < 0.05 was regarded as statistically significant.

## Results

### DDA1 expression profile in mouse tissue and lung cancer lines

**Figure 1:**
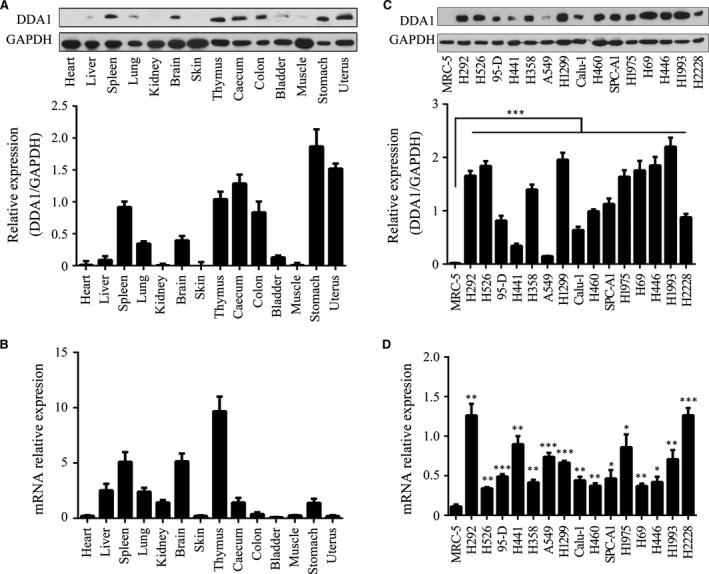
DDA1 expression profile in mouse tissue and different human lung cancer cell lines. (**A**) Upper panel: whole‐cell lysates of 6‐week‐old C57BL/6j mouse organs or tissue were analysed for DDA1 and GAPDH (as a loading control) protein expression by Western blot analysis. Lower panel: immunoblot densities of DDA1 and GAPDH were quantified using Image‐Pro Plus version 6.0 (Media Cybernetics, Inc. Rockville, MD, USA), and relative DDA1 expression *versus* GAPDH was determined (*n* = 3, data is shown as mean ± S.E.M.). (**B**) DDA1 expression and β‐actin mRNA expression in mouse tissue were quantified by qRT‐PCR, and the relative expression of DDA1 *versus* β‐actin is illustrated (*n* = 3, data are shown as mean ± S.E.M.). (**C**) Upper panel: whole‐cell lysates of normal lung fibroblast cells (MRC‐5) and 15 lung cancer cell lines were analysed for DDA1 and GAPDH protein expression by Western blot analysis. Lower panel: relative DDA1 expression *versus* GAPDH (*n* = 3, ***P < 0.001, Student's *t*‐test). (**D**) DDA1 expression and β‐actin mRNA expression in MRC‐5 and lung cancer lines were quantified by qRT‐PCR, and the relative DDA1 *versus* β‐actin levels are illustrated (*n* = 3, *P < 0.05, **P < 0.01, ***P < 0.001, Student's *t‐*test).

As a novel gene, the DDA1 expression profile had not been documented in mouse tissues and organs, previously. We analysed DDA1 protein and mRNA by Western blot and RT‐PCR, respectively, in 14 types of organs or tissues of C57BL/6j mouse under normal physiological conditions. We found that DDA1 expression was relatively lower in the lung and heart, while it was relatively higher in the spleen and thymus (Fig. 1A and B). We further wondered whether DDA1 was dysregulated in lung cancer cell lines compared to normal lung cells. Lung fibroblast MRC‐5 cells were used as a normal control, and 15 different lung cancer cell lines were assessed, and we found that both protein and mRNA were higher in cancer cell lines than in normal cells (Fig. 1C and D). These results indicate that the overexpression of DDA1 in lung cancer cells is common. Thus, DDA1 could be a molecular marker of lung cancer.

### DDA1 promotes cell proliferation and colony formation *in vitro*

**Figure 2:**
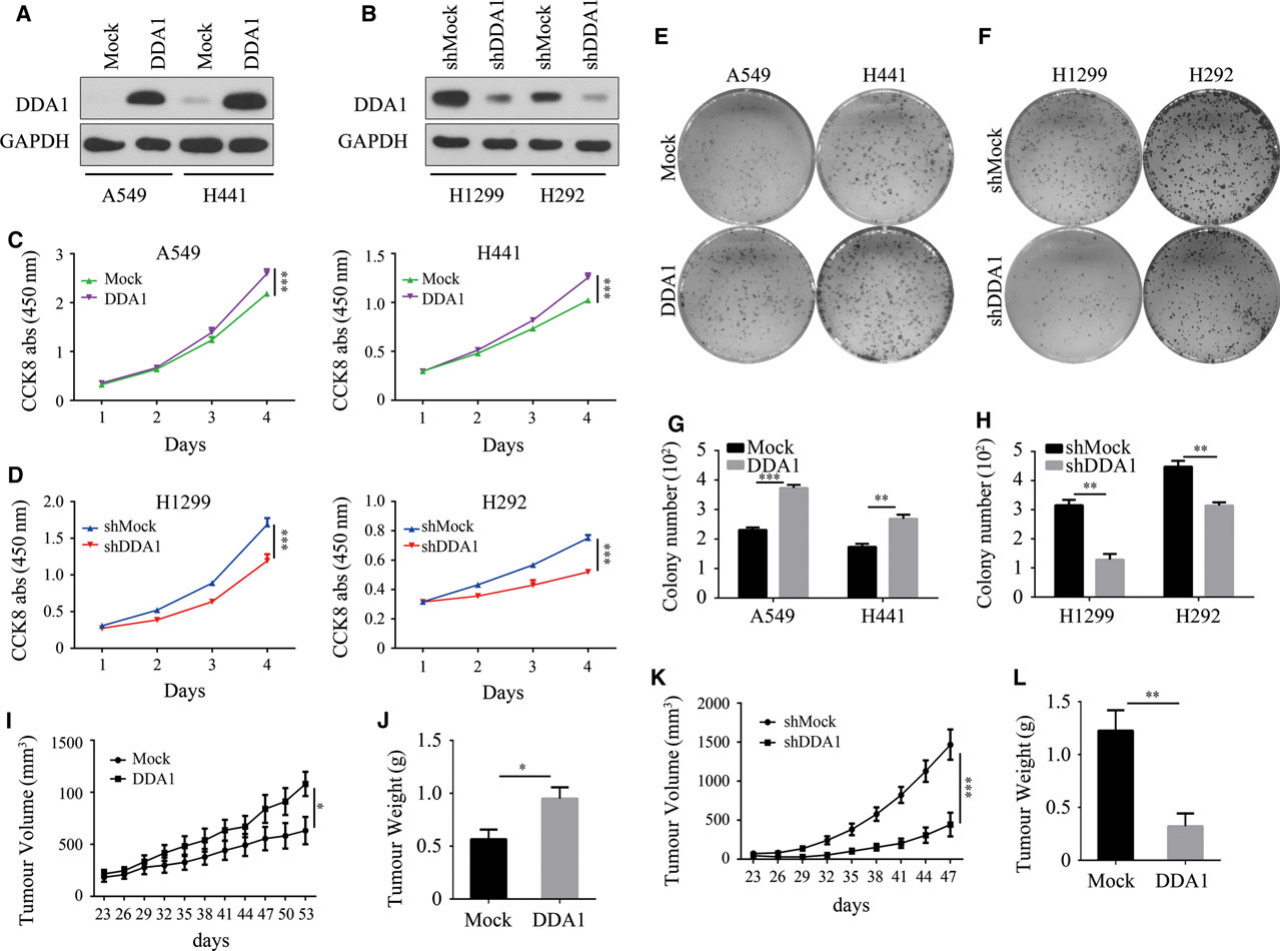
DDA1 promotes lung cancer cell proliferation *in vitro* and *in vivo*. (**A**) A549 and H441 cells were transfected and cultured for 48 hrs. Whole‐cell lysates were used to validate DDA1 and GAPDH (as a loading control) expression by Western blot analysis. (**B**) H1299 and H292 cells were transfected and then treated and analysed as in (**A**). (**C**) A549 and H441 cells were plated in 96‐well plates, and CCK8 measurements were performed every day as indicated (*n* = 6, ***P < 0.001, Student's *t‐*test). (**D**) H1299 and H292 cells were plated in 96‐well plate, and CCK8 measurements were performed every day as indicated (*n* = 6, ***P < 0.001, Student's *t‐*test). (**E**,** G**) A549 and H441 cells were subjected to colony formation assays in six‐well plates and cultured for 14 days followed by crystal violet staining. Colony number was calculated and analysed (*n* = 3, **P < 0.01, ***P < 0.001, Student's *t‐*test). (**F**,** H**) H1299 and H292 cells were subjected to colony formation assays as in (**E**,** G**) (*n* = 3, **P < 0.01, ***P < 0.001, Student's *t‐*test). (**I**,** J**) Tumour volume (*n* = 5, *P < 0.05, two‐way anova) and end‐stage tumour weight (*n* = 5, *P < 0.05, Student's *t‐*test) after injection of A549 cells stably transduced with lentivirus mock (Mock) or lentivirus DDA1 (DDA1) into nu/nu mice (*n* = 5). (**K**,** L**) Tumour volume (*n* = 5, ***P < 0.001, two‐way anova) and end‐stage tumour weight (*n* = 5, **P < 0.01, Student's *t*‐test) after injection of H1299 cells stably transduced with lentivirus shmock (shMock) or lentivirus shDDA1 (shDDA1) into nu/nu mice.

To explore the functional role of the DDA1 in regulating lung cancer development *in vitro*, DDA1 was overexpressed in A549 and H441 cells, which express endogenous DDA1 at a low level, through transfection with a plasmid encoding DDA1 (Fig. 2A). In addition, knockdown of DDA1 was achieved in H1299 and H292 cells, which express high levels of endogenous DDA1, through transfection with a plasmid expressing a short hairpin RNA targeting DDA1 (shDDA1) (Figs 2B and S1). Overexpression of DDA1 promoted cell proliferation significantly in both A549 (Mock: 2.181 ± 0.013 *versus* DDA1: 2.593 ± 0.044) and H441 (Mock: 1.021 ± 0.005 *versus* DDA1: 1.253 ± 0.022) cells (Fig. 2C); in contrast, inhibition of DDA1 reduced cell viability in both H1299 (shMock: 1.688 ± 0.043 *versus* shDDA1: 1.193 ± 0.045) and H292 (shMock: 0.751 ± 0.012 *versus* shDDA1: 0.520 ± 0.007) cells (Fig. 2D). Furthermore, the colony number increased significantly with up‐regulation of DDA1 in A549 (Mock: 231.0 ± 8.327 *versus* DDA1: 373.0 ± 10.69) and H441 (Mock: 173.7 ± 10.27 *versus* DDA1: 268.3 ± 14.40) cells (Fig. 2E and G), whereas colony formation of H1299 (shMock: 315.0 ± 18.93 *versus* shDDA1: 128.3 ± 19.65) and H292 (shMock: 447.7 ± 20.51 *versus* shDDA1: 314.0 ± 11.02) cells was hindered following inhibition of DDA1 expression (Fig. 2F and H). Taken together, DDA1, in lung cancer cells, determined the cell proliferative and colony formation abilities *in vitro*.

### DDA1 promotes tumour progression in a xenograft model

To explore whether the expression of DDA1 affects lung cancer growth *in vivo*, we transplanted A549 cells with or without DDA1 stable overexpression into nu/nu mice. Up‐regulation of DDA1 accelerated tumour growth *in vivo* (tumour volume: Mock: 631.6 ± 131.3 mm^3^ *versus* DDA1: 1080 ± 116.0 mm^3^, *n* = 5, *P* < 0.05; and tumour weight: Mock: 0.566 ± 0.091 g *versus* DDA1: 0.950 ± 0.106 g, *n* = 5, *P* < 0.05) (Fig. 2I and J). In contrast, we also transplanted H1299 cells with or without shRNA‐mediated stable knockdown of DDA1 into nu/nu mice. Deficiency in DDA1 significantly inhibited tumour growth, based on reductions in both tumour volume (shMock: 1468 ± 193.8 mm^3^ *versus* shDDA1: 443.6 ± 151.8 mm^3^, *n* = 5, *P* < 0.001) (Fig. 2K) and tumour weight (shMock: 1.226 ± 0.193 g *versus* shDDA1: 0.322 ± 0.121 g, *n* = 5, *P* < 0.01) (Fig. 2L). These results indicate that DDA1 promotes cancer cell proliferation *in vivo*.

### DDA1 affects cell cycle progression

**Figure 3:**
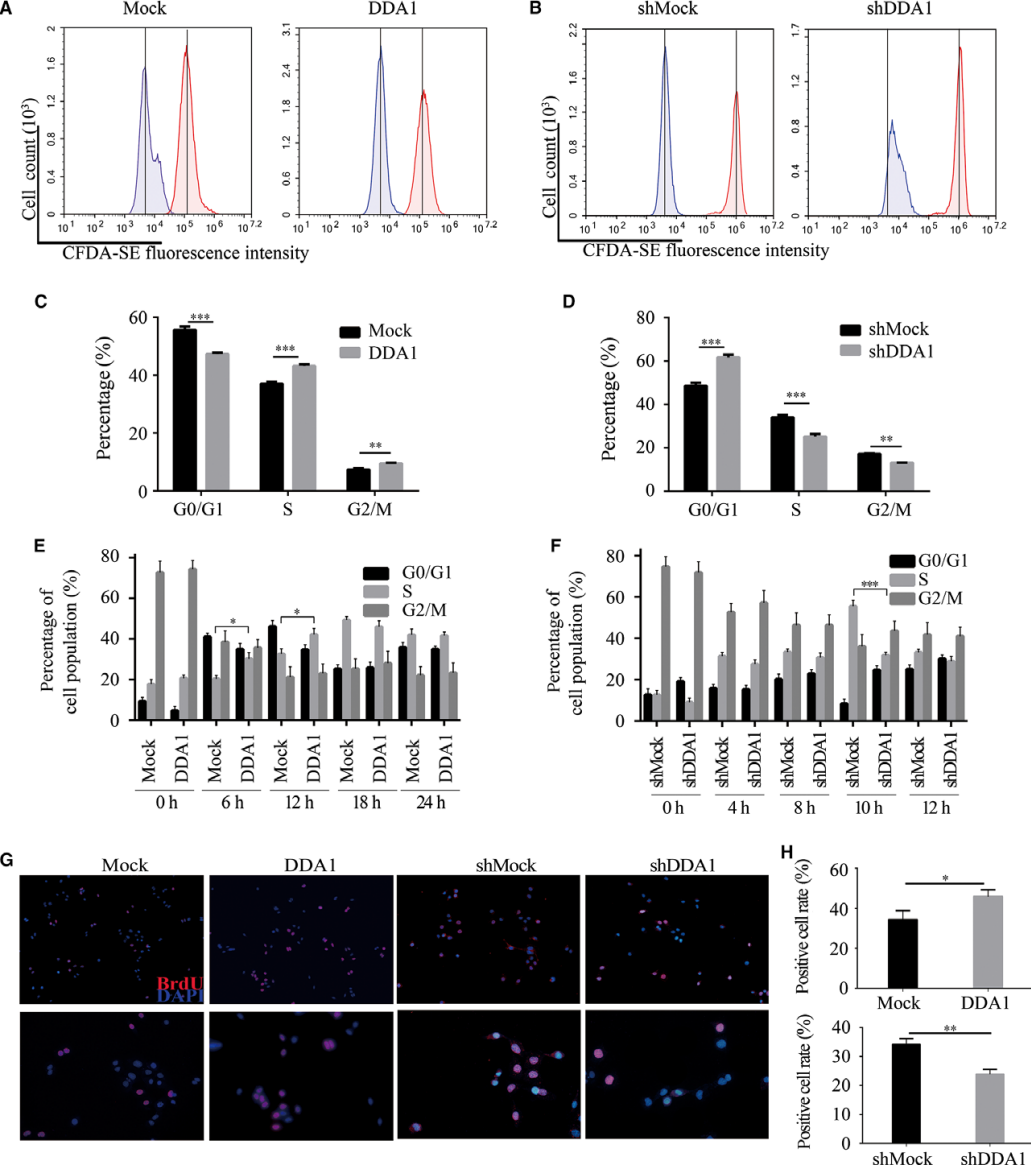
DDA1 affects cell cycle progression and G1/S transition. (**A**) A549 cells were transfected and cultured for 24 hrs followed by CFDA‐SE staining. CFDA‐SE fluorescence intensity was measured by flow cytometry after staining (0 days, red curve) or 4 days (blue curve). The vertical lines indicated the same values, respectively. (**B**) H1299 cells were transfected, treated and analysed as in (A). (**C**) A549 cells were transfected and cultured for 2 days followed by PI staining and flow cytometry for cell cycle analysis. Data were analysed by GraphPad Prism 6. The ratio of G0/G1 phase was decreased, and the proportion of cells in S and G2/M phases was significantly increased after overexpression of DDA1. (*n* = 3, **P < 0.01, ***P < 0.001, Student's *t*‐test). (**D**) H1299 cells were transfected and treated as in (**C**). The ratio of G0/G1 phase was increased, and proportion of cells in S and G2/M phases was significantly decreased after inhibition of DDA1. (*n* = 3, **P < 0.01, ***P < 0.001, Student's *t*‐test)**.** (**E**) A549 cells were transfected and cultured for 24 hrs followed by synchronization to G2/M phase by thymidine and nocodazole. The cells were released from blocking for indicated times and analysed by PI staining and flow cytometry. The proportion of S‐phase cells was significantly increased after 6 hrs. (*n* = 3, *P < 0.05, Student's *t*‐test)**.** (**F**) H1299 cells were transfected and treated as in (E). The percentage of S‐phase cells was decreased significantly after 10 hrs. (*n* = 3, ***P < 0.001, Student's *t*‐test)**.** (**G**,** H**) A549 and H1299 cells were transfected and cultured for 48 hrs. Cells were then probed by BrdU incorporation for 30 min. followed by anti‐BrdU antibody and DAPI staining. The BrdU‐positive cells were counted and analysed by GraphPad Prism 6. Data are presented as mean ± S.E.M. of three individual experiments. (*n* = 3, *P < 0.05, ***P < 0.001, Student's *t*‐test)**.**

To determine how DDA1 affects cancer cell prognosis and cell survival *in vitro*, we assessed the cell proliferation rate by CFDA‐SE (5‐(and‐6)‐carboxyfluorescein diacetate, succinimidyl ester) staining and flow cytometry. After staining with CFDA‐SE for 4 days, the fluorescence intensity of A549 cells overexpressing DDA1 was lower than that of control cells, indicating that DDA1 increased the frequency of cell division (Fig. 3A). In contrast, inhibition of DDA1 resulted in a higher proportion of cells exhibiting high fluorescence intensity, which suggested that H1299 cells grow slowly with ablation of DDA1 (Fig. 3B). As cell division is a direct reflection of cell cycle, we then assessed the proportion of cells in different cell cycle phases through PI staining and flow cytometry. We found that DDA1 overexpression resulted in a decreased proportion of G0/G1‐phase (Mock: 55.60 ± 0.69% *versus* DDA1: 47.33 ± 0.28%) and an increased percentage of S‐phase (Mock: 37.00 ± 0.46% *versus* DDA1: 43.20 ± 0.32%) cells (Figs 3C and S2A), and DDA1 knockdown resulted in an increased proportion of G0/G1‐phase (shMock: 48.70 ± 0.75% *versus* shDDA1: 61.67 ± 0.75%) and a decreased proportion of S‐phase (shMock: 34.03 ± 0.67% *versus* shDDA1: 25.20 ± 0.70%) cells (Figs 3D and S2B). Because changes in cell cycle are closely related to cell proliferation, these results suggest that DDA1 affects cell cycle progression resulting in cell proliferation.

### DDA1 promotes G1/S‐phase cell cycle transition

To examine the effect of DDA1 on different phases of cell cycle progression, we first assessed the cell cycle following thymidine and nocodazole synchronization. After synchronized to G2/M by thymidine and nocodazole, cells were transferred to normal cell culture medium for different durations and the G1/S transition to S‐phase ratio was assessed. We found that up‐regulation of DDA1 resulted in reducing the G1/S transition duration and an increased proportion of cells in S phase (6 hrs: Mock: 20.40 ± 1.74% *versus* DDA1: 30.30 ± 2.99%; 12 hrs: Mock: 32.60 ± 2.58% *versus* DDA1: 42.20 ± 2.99%) (Figs 3E and S3A) in A549 cells. Accordingly, knockdown of DDA1 in H1299 cells prolonged the G1/S transition and decreased the percentage of cells in S phase (10 hrs: shMock: 55.5 ± 2.87% *versus* shDDA1: 31.8 ± 1.52%) (Figs 3F and S3B). Furthermore, to examine the S‐phase kinetics in lung cancer cells, we monitored BrdU incorporation in asynchronized cells. Consistent with previous results, overexpression or knockdown of DDA1 increased (Mock: 34.3 ± 2.6% *versus* DDA1: 45.8 ± 2.0%) or decreased (shMock: 34.1 ± 1.2% *versus* shDDA1: 23.8 ± 1.0%) the proportion of BrdU‐positive (S‐phase) cells (Fig. 3G and H), respectively. These results suggest that DDA1 is responsible for G1/S‐phase transition and cell proliferation in lung cancer cells.

### DDA1 affects G2/M phase following G1/S transition

**Figure 4:**
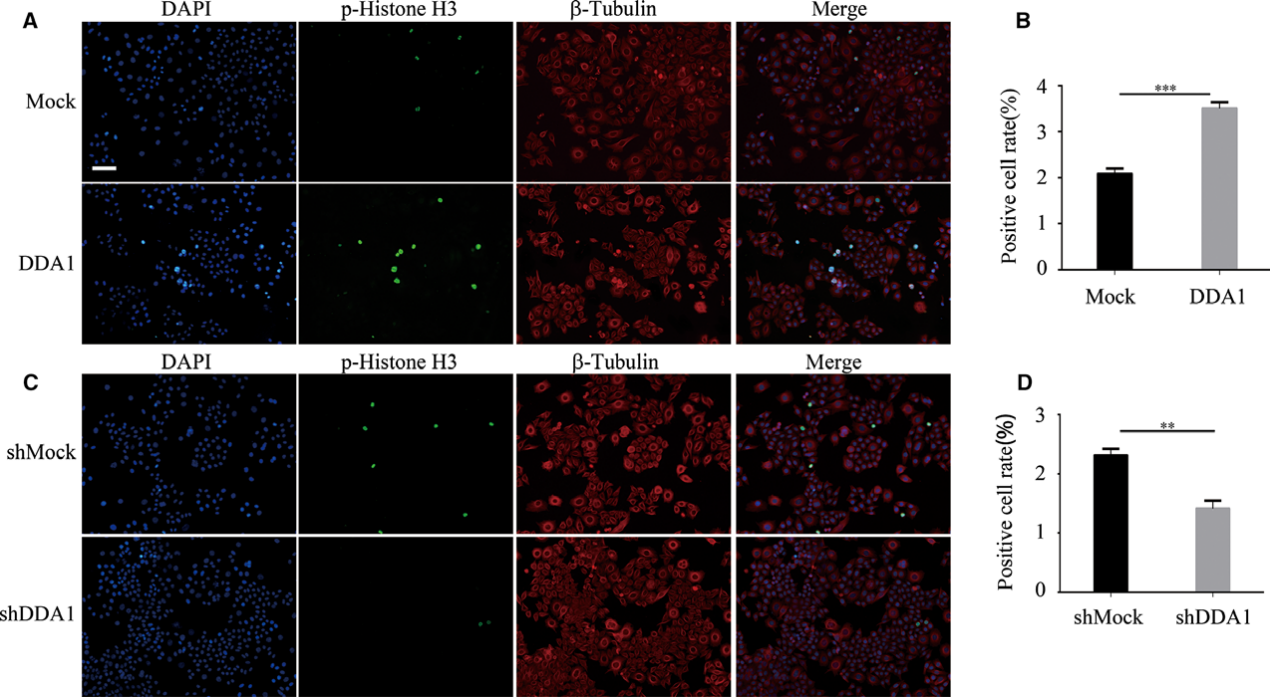
After S‐phase promotion, DDA1 also affected G2/M phase. (**A**,** B**) A549 cells were transfected and cultured for 48 hrs. Cells were then stained using anti‐phospho‐histone H3 (Ser10) (green) and β‐tubulin (red) antibodies and DAPI (blue). Phospho‐histone H3‐positive cells were counted and analysed by GraphPad Prism 6. (*n* = 3, ***P < 0.001, Student's *t*‐test). (**C**,** D**) H1299 cells were transfected and cultured for 48 hrs. Cells were then stained as in (**A**). Phospho‐histone H3‐positive cells were counted and analysed. Data are presented as mean ± S.E.M. of three individual experiments. (*n* = 3, **P < 0.01, Student's *t*‐test).

The median number of BrdU‐positive cells was altered after DDA1 up‐regulation or knockdown. As a result, the ratio of cells in the mitotic phase was affected, consistent with the fact that S‐phase cells yield M‐phase cells and that DDA1 altered the proportion of S‐phase cells. By measuring phospho‐histone H3 in these cells, we observed increased (Mock: 2.08 ± 0.07% *versus* DDA1: 3.50 ± 0.08%) or decreased (shMock: 2.31 ± 0.07% *versus* shDDA1: 1.41 ± 0.08%) mitotic entry with overexpression or silencing of DDA1 (Fig. 4A–D), respectively, in agreement with our previous results. These results are consistent with the hypothesis that lung cancer cells enter mitosis despite replication stress and that delayed mitotic entry is primarily a consequence of reduced replication.

### DDA1 promotes cell proliferation through regulating cyclins

**Figure 5:**
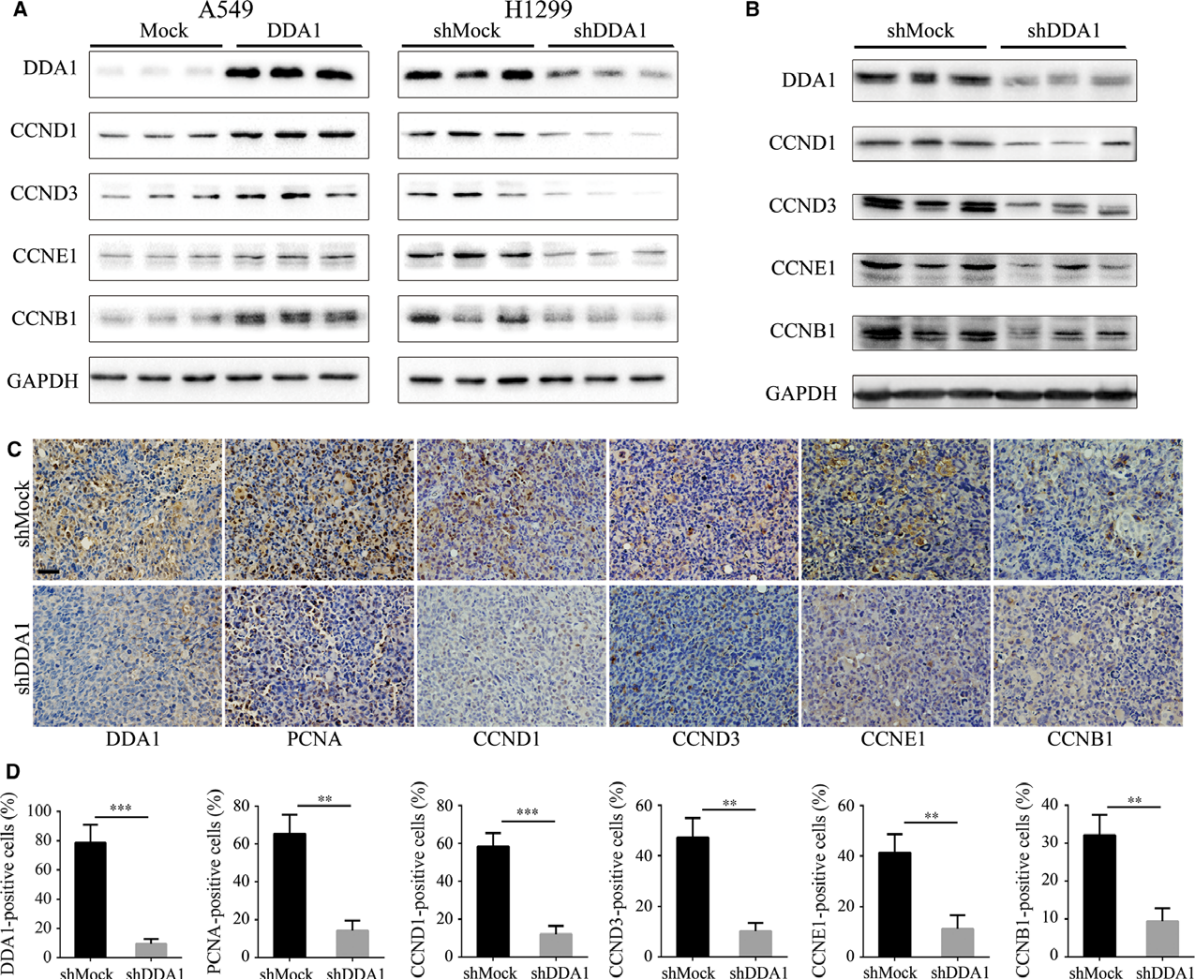
DDA1 promotes cell proliferation through the regulation of cyclins. (**A**) A549 and H1299 cells were transfected as indicated and cultured for 48 hrs. All treatments were performed for three duplications. Whole‐cell lysates were used to assess DDA1, CCND1, CCND3, CCNE1, CCNB1 and GAPDH (as a loading control) expression by Western blot analysis. (**B**) Whole tissue lysates from three independent tumours of DDA1‐deficient xenograft tumours from nude mice were used to evaluate DDA1, CCND1, CCND3, CCNE1, CCNB1 and GAPDH (as a loading control) expression by Western blot analysis. (**C**,** D**) Immunohistochemical probing for DDA1, PCNA, CCND1, CCND3, CCNE1 and CCNB1 using paraffin sections of DDA1‐deficient xenograft tumours from nude mice. Positive cells were counted by Image‐Pro Plus and analysed by GraphPad Prism 6. (*n* = 3, **P < 0.01, ***P < 0.001, Student's *t*‐test).

To explore the molecular mechanism of how DDA1 affects lung cancer, we suggested that DDA1 regulates the cyclins, which are the key regulators of cell cycle progression and proliferation. Overexpression or knockdown of DDA1 in lung cancer cells induced increase or decrease, respectively, in the expression of cyclin D1, cyclin D3, cyclin E1 and cyclin B1; increased expression of these cyclins is indicative of proliferation, G1 to S‐phase transition and subsequently M‐phase progression (Fig. 5A). In addition, similar results were observed in DDA1‐deficient (78.80 ± 6.99% *versus* 9.50 ± 1.85%) xenografts from nude mice (Fig. 5B). Furthermore, these xenografts had significantly lower PCNA (65.20 ± 5.95% *versus* 14.20 ± 3.12%), cyclin D1 (58.20 ± 4.22% *versus* 12.10 ± 2.48%), cyclin D3 (47.20 ± 4.50% *versus* 10.10 ± 1.91%), cyclin E1 (41.30 ± 4.22% *versus* 11.20 ± 3.12%) and cyclin B1 (32.10 ± 3.12% *versus* 9.40 ± 1.96%) levels than control tissues (Fig. 5C and D). These results indicate that DDA1 promotes tumour cell proliferation by regulating cell cycle‐associated cyclins.

### High expression of DDA1 correlates with lung cancer and poor prognosis

**Figure 6:**
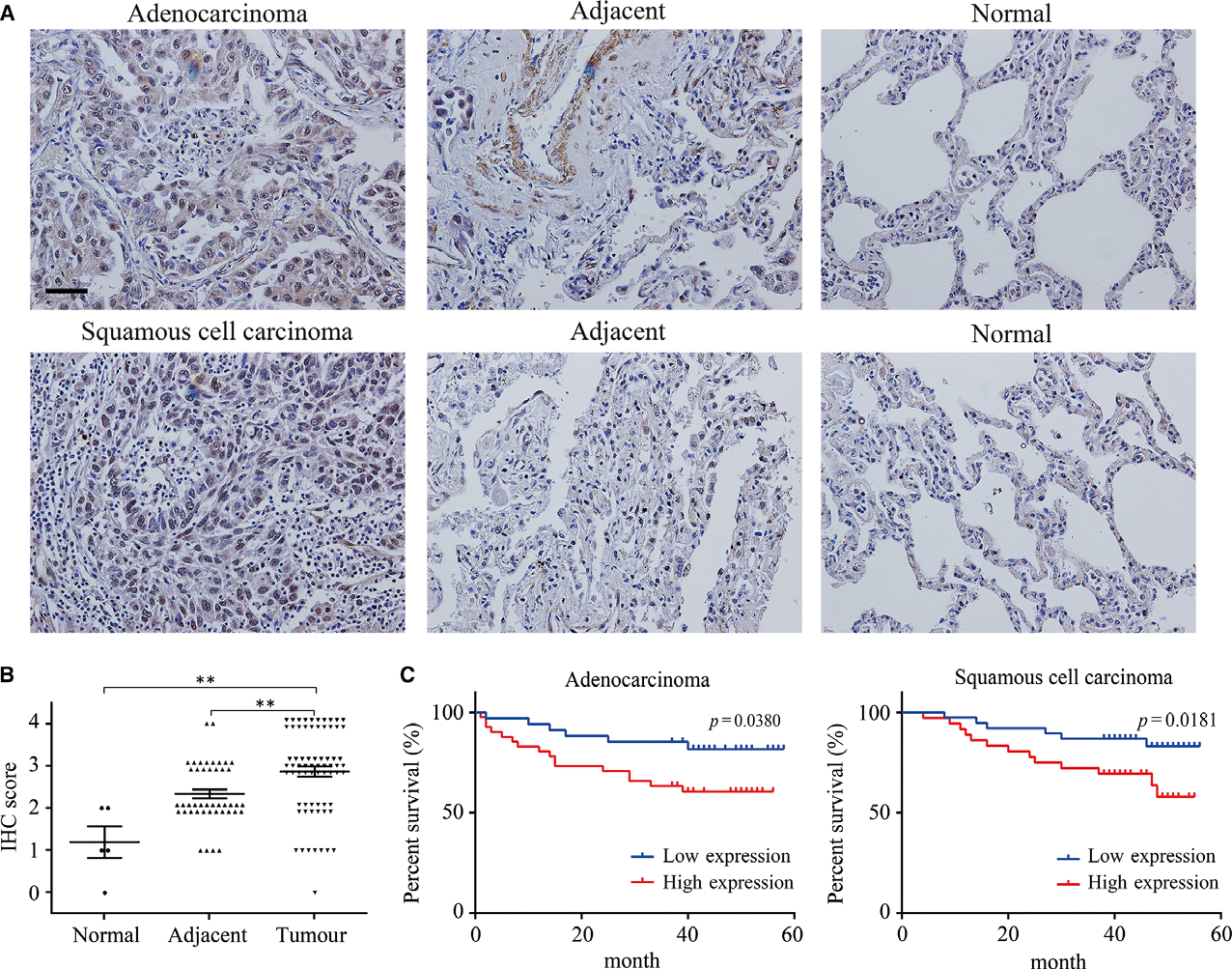
DDA1 is overexpressed in lung cancer tissue, and DDA1 expression correlates with shorter survival in lung cancer patients. (**A**) Analysis of tissue microarrays (TMAs). Immunohistochemical probing for DDA1 using TMAs containing 118 lung cancer, adjacent and normal tissue specimens (scale bar = 50 μm). (**B**) Statistical analysis was performed using GraphPad Prism 6 software to compare the relative levels of DDA1 among normal, adjacent and lung cancer tissues (nonparametric test, *n* = 118, *P* < 0.0001; Dunn's multiple comparison test, **P < 0.01). Black circle and triangles indicate the specimen, and the lines indicate the mean ± S.E.M. (**C**) Kaplan–Meier curve showing overall survival in patients (percentage) with lung cancer, stratified by DDA1 expression (high and low expression). DDA1 expression was determined by immunohistochemical staining and scored as described in the methods. The log‐rank test was used to compare differences between groups (*n* = 75; long‐rank test).

DDA1 promoted lung cancer tumorigenesis *in vitro* and *in vivo*; however, the relationship between DDA1 expression and human lung cancer was still unclear. To assess the expression of DDA1 in lung cancers, we performed immunohistochemistry (IHC) of DDA1 expression using a commercially available tissue microarray (TMA) containing 118 cases of lung cancer, adjacent tissue and normal tissue. Negative and positive staining of DDA1 in the epithelial cells was observed in the normal lung tissue, whereas DDA1 expression was high in lung cancer and the adjacent region, in most cases (Figs 6A and S4). DDA1 was expressed in the cytoplasm and nucleus, and its expression in cancer epithelial and stromal regions varied. To quantitate the protein expression of DDA1, we scored the staining intensity and proportion using five grades and found that DDA1 was significantly higher in lung cancer tissue and adjacent tissue than in normal tissue (**P < 0.01) (Figs 6B and S5). Next, we proceeded to determine whether overexpression of DDA1 is associated with prognosis. In both lung adenocarcinoma and squamous cell carcinoma, five‐year survival rates were much higher with low expression of DDA1 than with high expression, as assessed by the Kaplan–Meier method (*P < 0.05) (Fig. 6C).

The overexpression of DDA1 in lung cancer, based on positivity by IHC, was significantly associated with tumour size (*P* = 0.002) and AJCC stage (*P* = 0.016) (Table 1). However, the expression of DDA1 had no significant relationship with other clinical characteristics including gender, age, tumour type, positive LN number and histological grade.

**Table 1:** Correlations of DDA1 expression with clinical characteristics of lung tumour patients

Characteristics	Number of patients	DDA1 expression		*P* value
		Low	High	
Gender (%)				
Male	109	49	60	0.223
Female	41	23	18	
Age (%)				
≤60	70	37	33	0.292
>60	77	34	43	
Type (%)				
Ade	75	34	41	0.513
Squ	75	38	37	
Tumour size (%)				
≤5 cm	105	59	46	**0.002**
>5 cm	45	13	32	
Positive LN number (%)				
None	81	43	38	0.353
>0	64	29	35	
AJCC stage (%)				
I/II	77	42	35	**0.016**
>II	44	14	30	
Histological grade (%)				
I/II	114	55	59	0.915
>II	36	17	19	

A *P* value <0.05 was considered to indicate statistical significance. The *P* values were calculated in IBM SPSS Statistics 22.0 using chi‐square test.

The *p* value in bold indicate that the two group difference is statistical significant.

Univariate and multivariate Cox regression analyses were performed to evaluate the association between DDA1 and clinical factors, with prognostic hazards. Based on univariate Cox analysis, DDA1 (*P* = 0.003), tumour size (*P* = 0.012), positive LN number (*P* = 0.022) and AJCC stage (*P* < 0.001) were found to have an adverse impact on overall survival in patients with lung cancer (Table 2). Based on multivariate analysis, DDA1 (*P* = 0.005), tumour size (*P* = 0.012), positive LN number (*P* < 0.001) and AJCC stage (*P* < 0.001) were significant predictors of overall survival (Table 2). These results indicate that DDA1 is up‐regulated and positively correlated with poor prognosis of patients with lung cancer.

**Table 2:** Univariate and multivariate analysis of factors associated with overall survival of lung tumour patients

Factors	Univariate analysis		Multivariate analysis	
	Hazard ratio (95% CI)	*P* value	Hazard ratio (95% CI)	*P* value
DDA1	2.747 (1.405–5.372)	**0.003**		**0.005**
Gender	0.578 (0.267–1.248)	0.163		0.345
Age	1.586 (0.851–2.958)	0.147		0.309
Type	0.847 (0.462–1.552)	0.59		0.844
Tumour size	2.185 (1.189–4.015)	**0.012**		**0.012**
Positive LN number	2.094 (1.112–3.944)	**0.022**		**<0.001**
AJCC stage	4.42 (2.257–8.656)	**<0.001**	4.281 (2.186–8.384)	**<0.001**
Histological grade	1.142 (0.574–2.272)	0.706		0.996

Hazard ratios (95% confidence interval; CI) and *P* values were calculated using univariate or multivariate Cox proportional hazards regression in IBM SPSS Statistics 22.0. A *P* value <0.05 was considered to indicate statistical significance.

The *p* value in bold indicate that the two group difference is statistical significant.

## Discussion

Lung cancer is the leading cause of cancer‐related mortality. An understanding of the mechanisms through which tumours initiate, progress, metastasize or acquire resistance to targeted therapies is critical for the identification of additional pathways that can be targeted and for predicting which patients will respond to specific therapies to maximize clinical benefits 21. EGFR, KRAS and EML4‐ALK are well‐understood oncogenic drivers of lung cancer 22, 23, 24, and patients with lung tumours that are dependent on these oncogenes are treated with EGFR inhibitors, KRAS pathway inhibitors (antroquinonol or AZD6244) and ALK inhibitors (crizotinib), as well as newer second‐generation inhibitors 25, 26. As responses to these agents are generally short‐lived, an understanding of other mechanisms leading to lung cancer is essential for the design of therapeutic strategies to improve efficacy. We found that enhanced DDA1 expression is common in human lung cancer. Overexpression of DDA1 was observed in most lung cancer samples and all lung cancer cell lines investigated. Our results also indicated that DDA1 is up‐regulated and positively correlated with poor prognosis in patients with lung cancer. DDA1 could thus be a molecular marker of lung cancer.

Loss of growth control is a hallmark of cancer 9. Cell cycle and cell growth are governed by two distinct levels of regulation. One is the transcriptional control of genes 27, and the other is the degradation of proteins involved in chromosomal DNA replication and segregation 28. Therefore, cell cycle phases, particularly G1 and G2, are not defined by the content of genomic DNA alone, but also by the status of the above two systems 29. In the present study, DDA1 was shown to be responsible for lung cancer cell G1/S transition and cell proliferation through the regulation of cyclin D1, cyclin D3, cyclin E1 and cyclin B1 expression. The proliferation of mammalian cells is driven by cyclins, which are key components of the cell cycle machinery that bind to, activate and provide substrate specificity for CDKs, thereby driving cell cycle progression 30. Cyclin D1 and cyclin D3 are dysregulated in a variety of human cancers, and the inhibition of these proteins represents a highly selective anticancer strategy that specifically targets cancer cells without significantly affecting normal tissues 15, 18, 31, 32, 33. Cyclin E1 overexpression correlates with poor overall survival in several tumour types including breast, ovarian, lung and pancreatic cancer 34, 35. Attenuation of the PI3K/PKCiota/cyclin E1 pathway is a target in ovarian cancer 36. Deregulation of cyclin E1 causes human mammary epithelial cells to enter mitosis with short unreplicated genomic segments at a small number of specific loci, leading to anaphase anomalies and ultimately deletions 37. The fundamental role of cyclin B1/Cdk1 is in the regulation of cytoplasmic and nuclear events required for G2/M transitions. Cyclin B1/Cdk1 might also function in co‐ordinating mitochondrial bioenergetics for cell cycle G2/M progression 38. Recently, Aregger *et al*. 39 found that RNMT is phosphorylated and activated by CDK1‐cyclin B1, resulting in elevated cap methyltransferase activity at the beginning of the G1 phase. DDA1 could regulate these cyclins leading to manipulation the G1/S transition and cell proliferation.

DDA1, together with DET1 and DDB1, might be involved in ubiquitination and subsequent proteasomal degradation of target proteins 2, 4. Moreover, DDA1 was demonstrated to be a core subunit of multiple CRLs and might regulate CRL4s 5.

CRL4s are involved in the regulation of the DNA damage response, histone modification and nucleosome assembly 40, 41, 42. Knockdown of Cul4A results in chromatin dysfunctions in yeast and mammalian cells, and Cul4A is overexpressed in many cancer types 43. CRL4s are also reported to have important functions in the cell cycle, especially in S‐phase‐related protein degradation. CRL4s have been shown to target several key factors including Cdt1, p21, p27, E2f1 and Chk1 for degradation, to maintain proper S‐phase and S‐G2 progression 41, 43, 44, 45. DDB1, as the linker protein for the Cul4 E3 ubiquitin ligase, regulates proteins that are essential for nucleotide excision repair, cell cycle progression, DNA replication and cell growth 46. Future studies are required to elucidate the molecular mechanism through which DDA1 regulates cyclin expression and cell cycle progression.

In the study, we found that both protein and mRNA were higher in cancer cell lines than in normal cells, while DDA1 mRNA was not positively associated with protein level and the mechanism was still unclear. The cellular proteome is a complex microcosm of structural and regulatory networks that requires continuous surveillance and modification to meet the dynamic needs of the cell 47. Genetic alterations, including chromosome alteration, oncogene activation, miRNA dysregulation, mRNA decay, autophagy and ubiquitin–proteasome system 47, 48, can affect protein biogenesis and degradation systems, which often results in proteome imbalance. mRNA half‐lives differ significantly between various transcripts in all eukaryotic organisms investigated so far 49, 50. Interestingly, decay rates for some mRNAs seem to be conserved between different species to some extent 51. Notably, mRNAs encoding housekeeping proteins tend to have considerably longer half‐lives than those encoding regulatory proteins 52. Connexin 31.1 was newly reported to be down‐regulated in NSCLC cell line through both ubiquitin–proteasome system (UPS) and autophagy 53. So, further investigations on the regulatory mechanism should be needed in the future.

In summary, in this study, we found that DDA1 is commonly up‐regulated in lung cancer tissue and cell lines and that higher expression level of DDA1 is associated with poor prognosis in patients with lung cancer. The results *in vitro* show that the DDA1 enhances proliferation in tumour cells and promotes S‐phase entry through the regulation of cell cycle‐related proteins. *In vivo* studies determined that overexpression of DDA1 can promote the tumour growth, while inhibition of DDA1 gene expression can significantly inhibit the tumour growth in subcutaneous xenograft model. DDA1 as an oncogenic factor could be a target of future therapeutics or could be applied as a predictive marker of lung cancer. Further evaluation of DDA1 as a target for lung cancer therapy and the molecular mechanism is required using additional preclinical animal models and other approaches. In addition, the predictive value of DDA1 should be expanded to larger patient cohort studies.

## Author contributions

H. Deng and L. Cheng conceived the project and supervised research. C. Li and Y. Yang prepared the manuscript. L. Dai, L. Liu and J. Zhang provided the original data. L. Cheng, Q. Yang and Q. Wang designed and performed the experiments. Y. Ding, P. Fan and X. Hu collected the clinical samples. S. Zhang and R. Xiang helped to perform statistical analysis. Y. Wei and D. Yu contributed essential reagents or tools. All authors reviewed the manuscript.

## Conflict of interest

The authors confirm that there are no conflicts of interest.

## Supporting information


**Figure S1** Interference effect of shRNA targeting DDA1. H1299 cells was transfected and cultured for 48 hrs followed by western blot analysis of the whole cell lysates
**Figure S2** (**A**) A549 cells were transfected and cultured for 2 days followed by PI staining and flow cytometry for cell cycle analysis. The ratio of G0/G1 phase was decreased and the proportion of cells in S and G2/M phases was significantly increased after overexpression of DDA1. (**B**) H1299 cells were transfected and cultured for 2 days followed by PI staining and flow cytometry for cell cycle. The ratio of G0/G1 phase was increased and proportion of cells in S and G2/M phases was significantly decreased after inhibition of DDA1
**Figure S3** (**A**) A549 cells were transfected and cultured for 24 hrs followed by synchronization to G2/M phase by thymidine and nocodazole. The cells were released from blocking for indicated times and analyzed by PI staining and flow cytometry. The proportion of S‐phase cells was significantly increased after 6 hrs (**B**) H1299 cells were transfected and treated as in (**A**). Then cells were released from blocking for indicated times and analyzed by PI staining and flow cytometry. The percentage of S‐phase cells was decreased significantly after 10 hrs
**Figure S4** DDA1 is overexpressed in lung cancer tissue. 8 pairs of tumor (T) and normal (N) tissue of lung cancer patients were assessed by western blot and DDA1 level in all these tumor tissues was higher than that of normal tissues
**Figure S5** Representative IHC score of TMA tissue sections
**Table S1** shRNA sequence of DDA1
**Table S2** Primers used for qPCR
