# Supplementary material for: DDA1, a novel oncogene, promotes lung cancer progression through regulation of cell cycle
Source: J Cell Mol Med. 2017 Feb 17;21(8):1532–44. doi: 10.1111/jcmm.13084 (PMC5542901; doi:10.1111/jcmm.13084)
Supplement: Supplementary file 1 — Figure S1 Interference effect of shRNA targeting DDA1. H1299 cells was transfected and cultured for 48 hrs followed by western blot analysis of the whole cell lysates Figure S2 (A) A549 cells were transfected and cultured for 2 days followed by PI staining and flow cytometry for cell cycle analysis. The ratio of G0/G1 phase was decreased and the proportion of cells in S and G2/M phases was significantly increased after overexpression of DDA1. (B) H1299 cells were transfected and cultured for 2 days followed by PI staining and flow cytometry for cell cycle. The ratio of G0/G1 phase was increased and proportion of cells in S and G2/M phases was significantly decreased after inhibition of DDA1 Figure S3 (A) A549 cells were transfected and cultured for 24 hrs followed by synchronization to G2/M phase by thymidine and nocodazole. The cells were released from blocking for indicated times and analyzed by PI staining and flow cytometry. The proportion of S‐phase cells was significantly increased after 6 hrs (B) H1299 cells were transfected and treated as in (A). Then cells were released from blocking for indicated times and analyzed by PI staining and flow cytometry. The percentage of S‐phase cells was decreased significantly after 10 hrs Figure S4 DDA1 is overexpressed in lung cancer tissue. 8 pairs of tumor (T) and normal (N) tissue of lung cancer patients were assessed by western blot and DDA1 level in all these tumor tissues was higher than that of normal tissues Figure S5 Representative IHC score of TMA tissue sections Table S1 shRNA sequence of DDA1 Table S2 Primers used for qPCR [file JCMM-21-1532-s001.doc]

**Supplemental Figures**

**
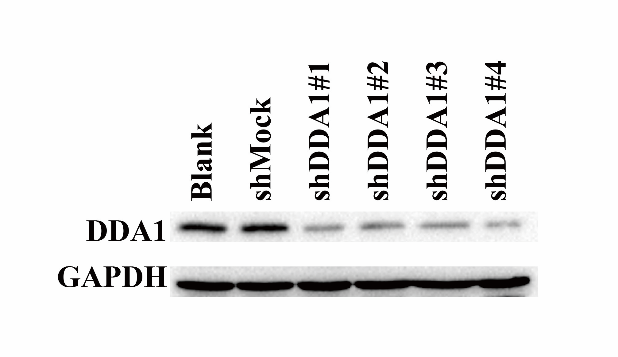
**

Fig S1. Interference effect of shRNA targeting DDA1. H1299 cells was transfected and cultured for 48 hours followed by western blot analysis of the whole cell lysates.


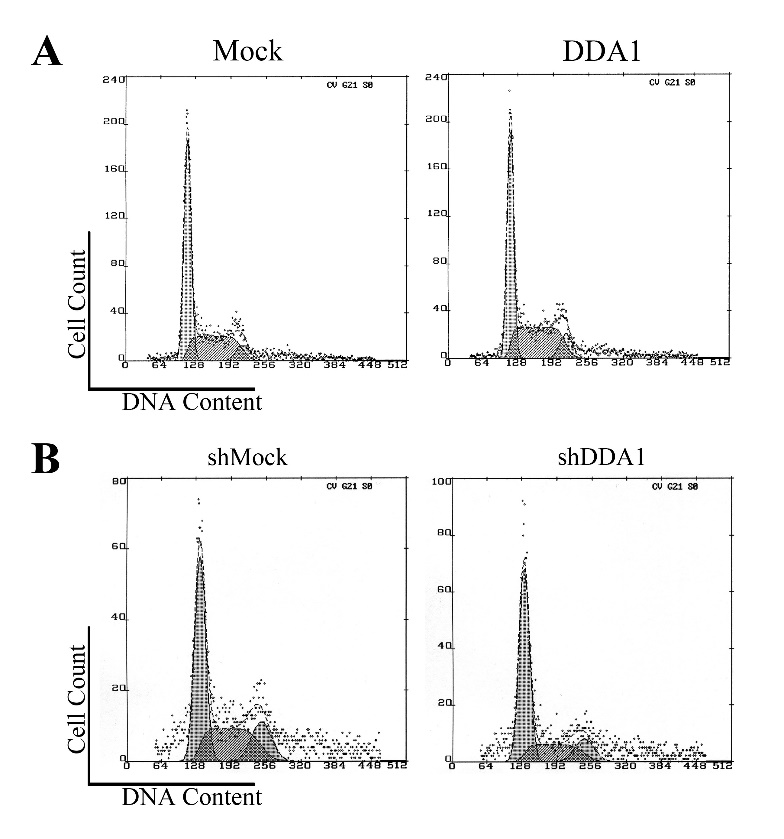


Fig S2. (A) A549 cells were transfected and cultured for 2 days followed by PI staining and flow cytometry for cell cycle analysis. The ratio of G0/G1 phase was decreased and the proportion of cells in S and G2/M phases was significantly increased after overexpression of DDA1. (B) H1299 cells were transfected and cultured for 2 days followed by PI staining and flow cytometry for cell cycle. The ratio of G0/G1 phase was increased and proportion of cells in S and G2/M phases was significantly decreased after inhibition of DDA1.


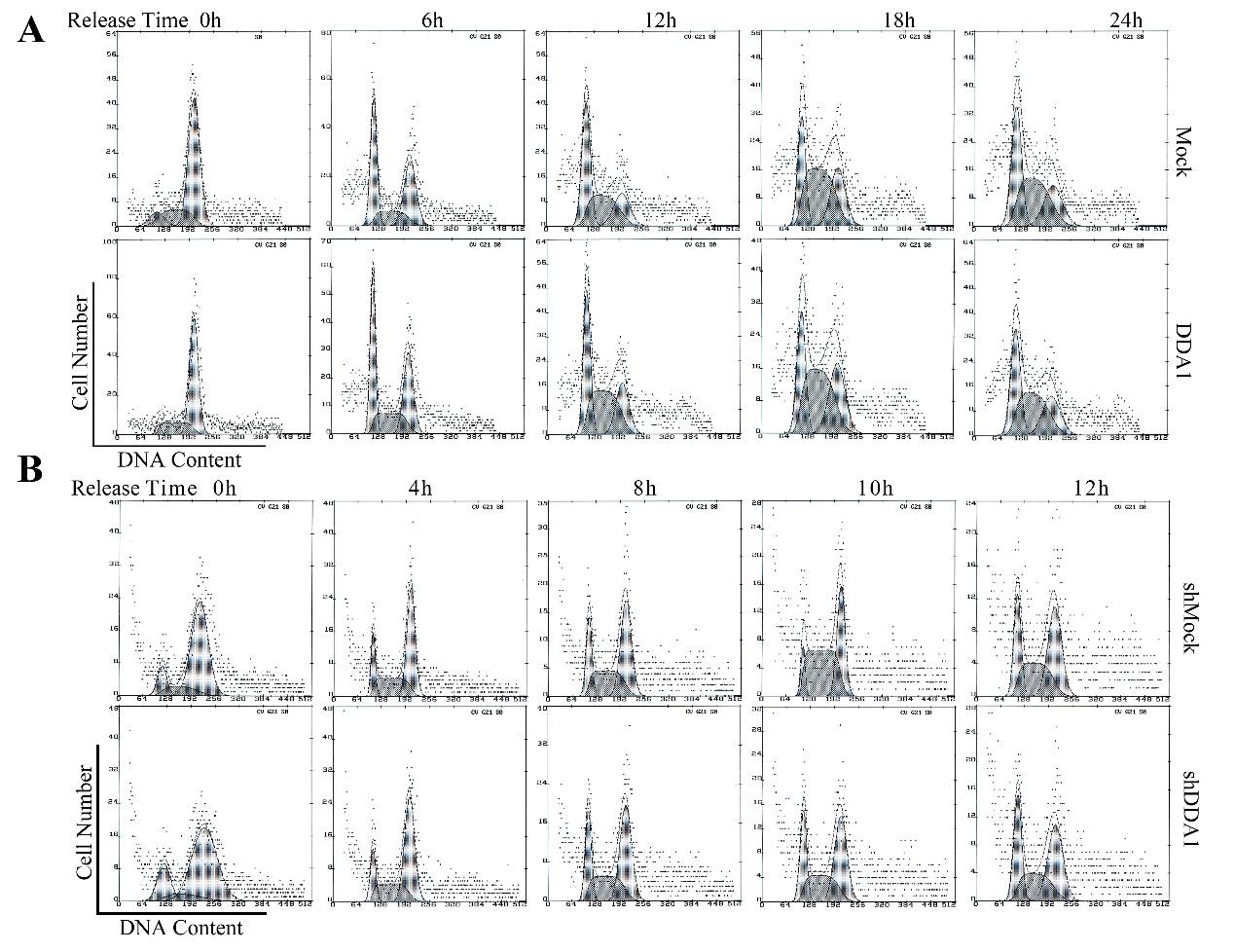


Fig S3. (A) A549 cells were transfected and cultured for 24 hours followed by synchronization to G2/M phase by thymidine and nocodazole. The cells were released from blocking for indicated times and analyzed by PI staining and flow cytometry. The proportion of S-phase cells was significantly increased after 6 hours (B) H1299 cells were transfected and treated as in (A). Then cells were released from blocking for indicated times and analyzed by PI staining and flow cytometry. The percentage of S-phase cells was decreased significantly after 10 hours.


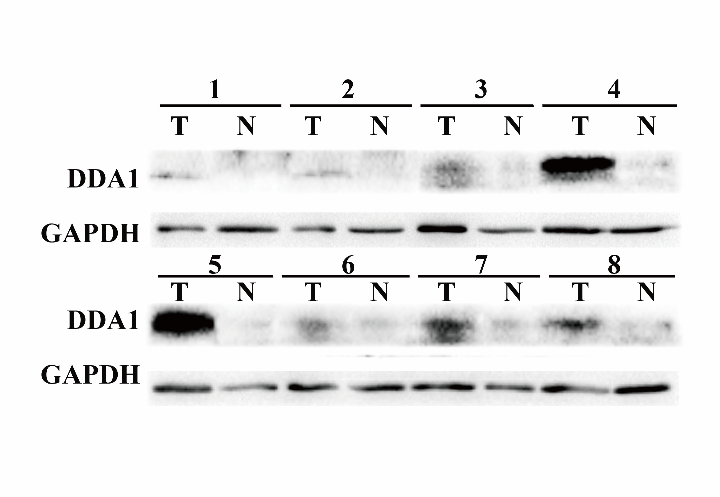


Fig S4. DDA1 is overexpressed in lung cancer tissue. 8 pairs of tumor (T) and normal (N) tissue of lung cancer patients were assessed by western blot and DDA1 level in all these tumor tissues was higher than that of normal tissues.


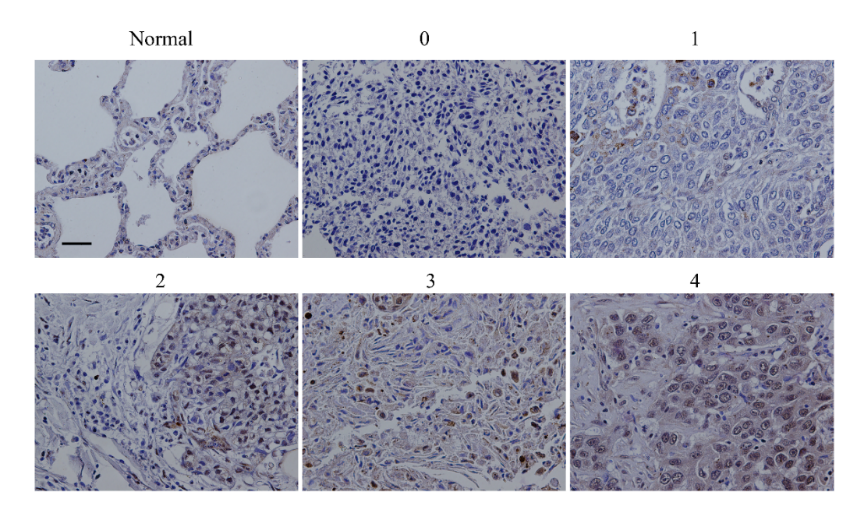


Fig S5. Representative IHC score of TMA tissue sections.

**Supplemental Materials**

Table S1. shRNA sequence of DDA1.

| Symbol | Seqence (5'-3') |
| --- | --- |
| #1-257 | GAACAGATCATCGTGACAGAA |
| #2-2781 | CCTCATAGGAGCCGATGTATT |
| #3-337 | GCGGTGTCCATCTGTGAATGA |
| #4-571 | GAAGAGAGACCAGGAGCAAGT |

Table S2. Primers used for qPCR.

| Organism | Gene | Type | Sequence (5'-3') |
| --- | --- | --- | --- |
| Human | DDA1 | Forward | GCCCTCAGTCTACCTGCCTA |
|  |  | Reverse | TCCTGGTCTCTCTTCTTGGC |
|  | β-actin | Forward | TGGACTTCGAGCAAGAGATG |
|  |  | Reverse | GAAGGAAGGCTGGAAGAGTG |
| Mouse | DDA1 | Forward | CTTGCCCGTCTACAACAAGAG |
|  |  | Reverse | GCAGGTACCGCAGAAGTATG |
|  | β-actin | Forward | GTACTCTGTGTGGATCGGTGG |
|  |  | Reverse | GCAGCTCAGTAACAGTCCG |

**Antibody used in this study.**

Rabbit polyclonal antibody against DDA1 (Cat 14995-1-AP, ProteinTech, Chicago, IL, USA); Rabbit monoclonal antibody against GAPDH (Cat 60004-1-Ig, ProteinTech, Chicago, IL, USA); mouse monoclonal antibody against BrdU (Bu20a), (Cat #5292, Cell Signalling, Danvers, MA, USA); mouse monoclonal antibody against β-Tubulin(Clone AA2) (Cat T8328, Sigma-aldrich, St. Louis, MO, USA); rabbit monoclonal antibody against Cyclin D1(92G2), and Phospho-Histone H3 (Ser10) (D2C8) (Cat #2978 and #3377, Cell Signalling, Danvers, MA, USA); rabbit polyclonal antibody against Cyclin B1 (Cat #4138, Cell Signalling, Danvers, MA, USA); rabbit monoclonal antibody against Cyclin D3 (EP43E) (Cat ab52598, Abcam, Cambridge, MA, USA.); rabbit polyclonal antibody against Cyclin E (Cat ARG70003, Arigo, Acton, MA, USA); mouse monoclonal antibody against PCNA (PC10), (Cat sc-56, Santa Cruz, Dallas, Texas, USA); goat anti-rabbit IgG-FITC and goat anti-mouse IgG-TR (Cat sc-2012 and sc-2781, Santa Cruz, Dallas, Texas, USA).
